# Nail or plate in the management of distal extra-articular tibial fracture, what is better? Valutation of outcomes

**DOI:** 10.1051/sicotj/2017058

**Published:** 2018-02-21

**Authors:** Michele Bisaccia, Andrea Cappiello, Luigi Meccariello, Giuseppe Rinonapoli, Gabriele Falzarano, Antonio Medici, Cristina Ibáñez Vicente, Luigi Piscitelli, Verdiana Stano, Olga Bisaccia, Auro Caraffa

**Affiliations:** 1 Department of Orthopaedics and Traumatology, “S.M. Misericordia Hospital”, University of Perugia, Perugia Italy; 2 Department of Orthopedics and Traumatology, Vito Fazzi Hospital, Lecce Italy; 3 Department of Medical and Surgical Sciences and Neuroscience, Section of Orthopedics and Traumatology, University of Siena, University Hospital “Santa Maria alle Scotte”, Siena Italy; 4 Department of Civil Engineering and Computer Engineering, Faculty of Medical Engineering, University of Rome Tor Vergata, Rome Italy; 5 Department of Radiology, “San Donato Hospital” University of Milano, Milano Italy

**Keywords:** Plate, Nail, Extra-articular distal tibia, Outcome, Surgical management distal tibia

## Abstract

*Introduction*: Distal tibial fractures are the most common long bone fractures. Several studies focusing on the methods of treatment of displaced distal tibial fractures have been published. To date, locked plates, intramedullary nails and external fixation are the three most used techniques. The aim of our study was to compare intramedullary nail (IMN) and locked plate (LP) for treatment of this kind of fracture.

*Materials and methods*: We collected data on 81 patients with distal tibial fractures (distance from the joint between 40 and 100 mm) and we divided into two groups: IMN and LP. We compared in the 2 groups the mean operation time, the mean union time, the infection rate the rate of malunion and nonunion, the full weight bearing time.

*Results*: No patient in the two groups developed a nonunion. None of the patients obtained a fair or poor outcome. Overall 52 patients obtained an excellent result (69.3%) and 23 obtained a good result (30.6%).

*Discussion*: Our study results indicate a superiority of IMN over LP in terms of lower rates of infections and statistically significant shorter time to full weight bearing. Whereas LP appeared to be advantageous over IMN in terms of leading to a better anatomical and fixed reductions of the fracture and a lower rate of union complications. The two treatments achieved comparable results in terms of operation time, hospital stay, union time and functional outcomes.

## Introduction

Distal tibial fractures are the most common long bone fractures. Published data suggest an incidence of 17 per 100 000 person-years [1], although more recent data indicate that the incidence may be declining [2]. In most cases, they are due to a force directed from the foot towards the leg in the environment of outstanding high-energy traumatic events, as fall down, traffic accident, motorcycle accident or sport injury [3,4].

Their management presents a series of problems because this kind of fractures could determine the damage of the surrounding soft tissues; indeed, soft tissues are very thin in this region of the leg; furthermore, tibial distal fractures are even more at risk of exposure because of their proximity to the ankle and the lack of arterial supply in the distal tibia [4,5]. In a rate of 80% of this kind of traumas, fibula is involved. Furthermore, fibula tends to heal more rapidly than tibia [1].

Several studies focusing on the methods of treatment of displaced distal tibial fractures have been published [5–11]. To date, locked plates, intramedullary nails and external fixation are the three most used techniques, but each has been historically related to complications: mal-alignment and knee pain have been associated with nailing; infections, wound complications and implant prominence are frequently reported after tibial plating; prolonged fracture healing, frequent need of secondary operations and infections of the pin tract are inherent problems in external fixation [6,7,12].

The aim of our study was to compare intramedullary nail (IMN) and locked plate (LP) for: operation time, hospital stay, union time, rate of infections and union complications (nonunion and malunion).

## Materials and methods

**Figure 1:**
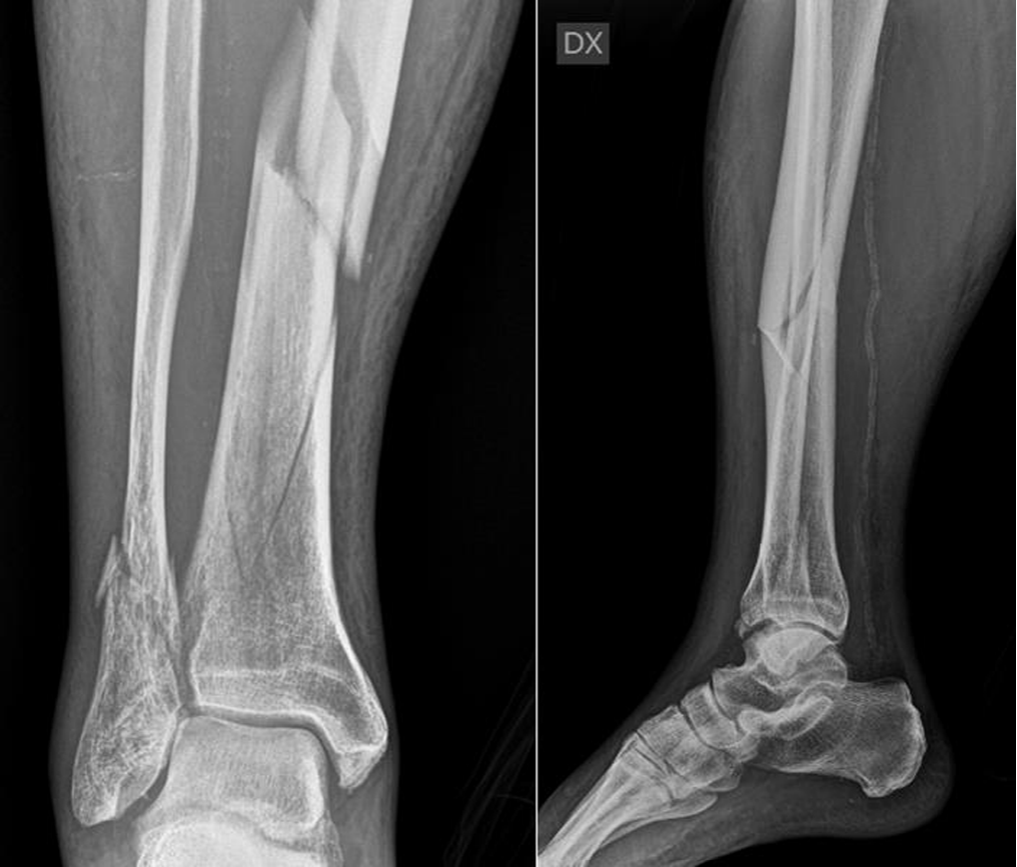
Tibial distal fracture AO 42-B1 with irradiation of the fracture line up to the joint.

**Figure 2:**
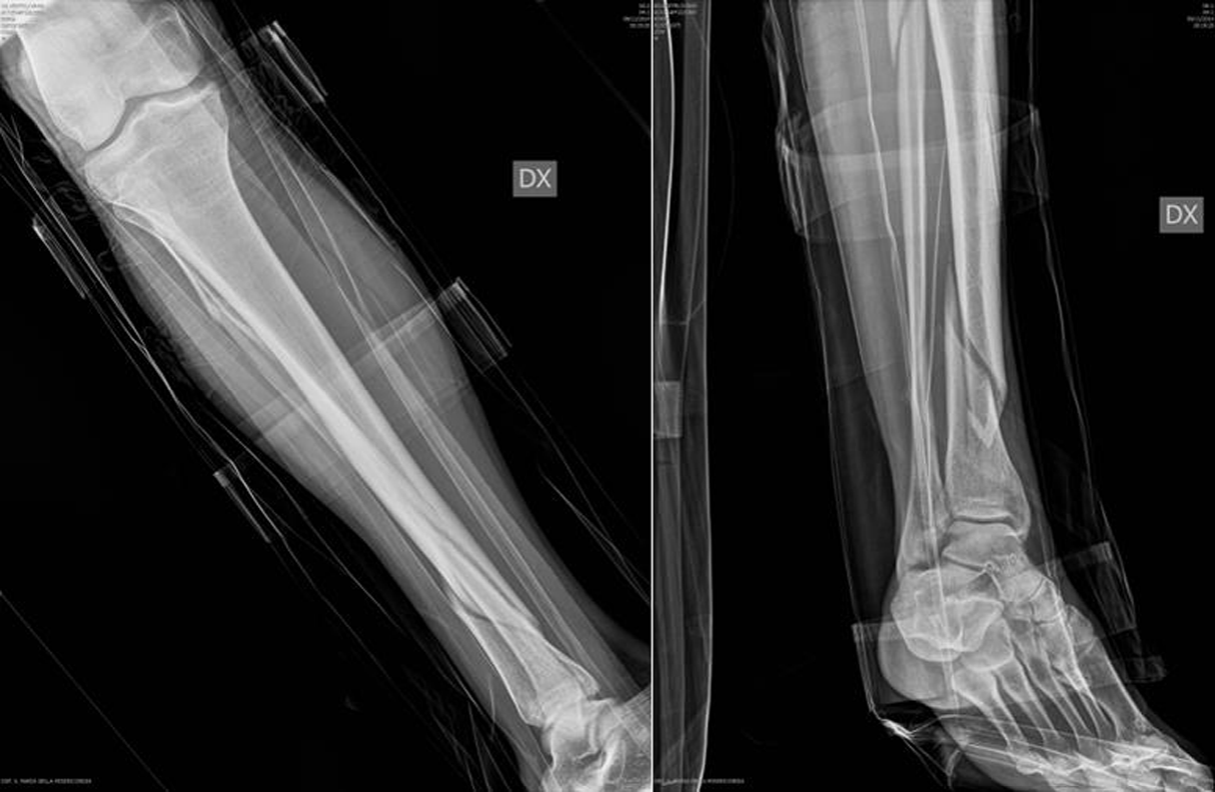
(a,b) AO: 42-A1 fracture of right tibia.

We collected our data retrospectively between January 2009 and January 2015, on 81 patients with distal tibial fractures (Figures 1, 2a and b).

**Figure 3:**
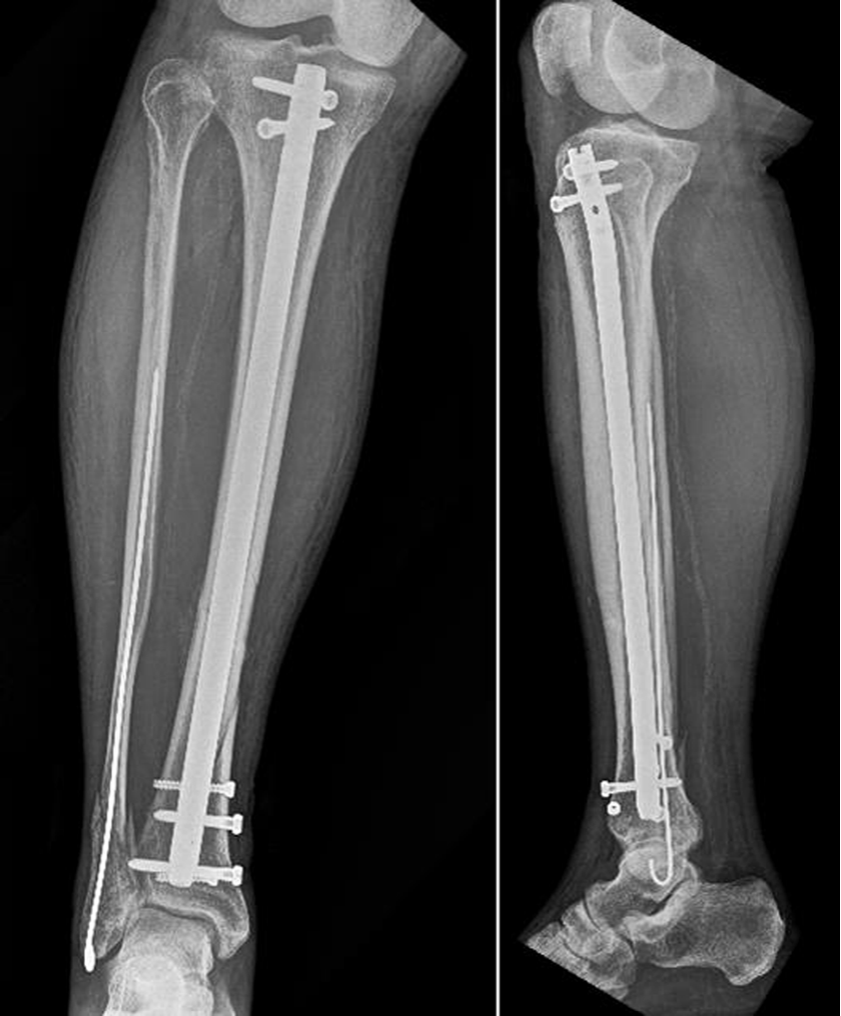
The same fracture of Figure 1 after surgery. IMN and K wire to stabilize the fibua. Use of Poller's screws in the distal tibia because the fracture was very close to joint line.

**Figure 4:**
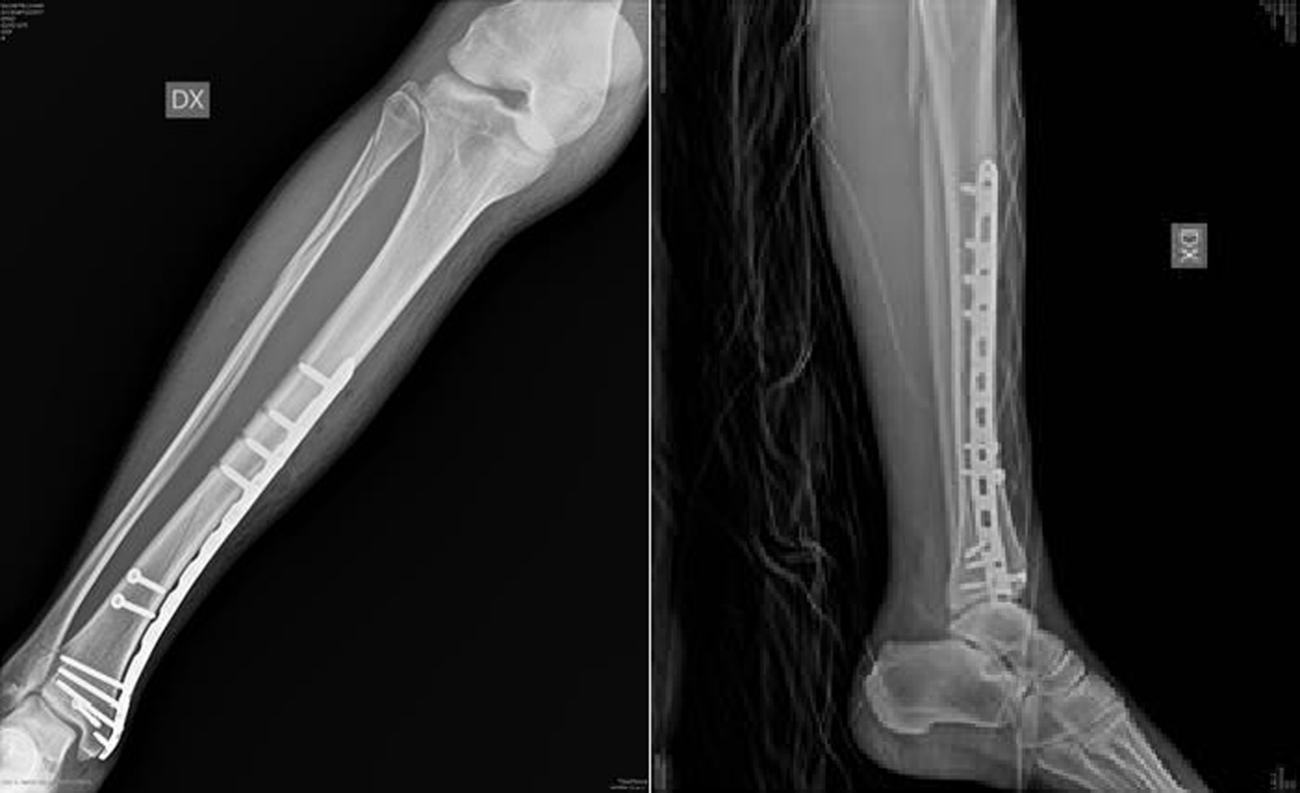
(a,b) The same case of Figure 2 after surgery. We use a LCP plate for distal tibia and 3 lag screws.

Patients were divided into two groups: the IMN group included 41 patients who underwent IMN (Figure 3), whereas LP group included 34 patients who underwent LP (Figures 4a and b).

The distance from the joint was in all the cases between 40 and 100 mm. All the tibial fractures were associated with fibular fractures. In 54 patients we performed a stabilization with intramedullary K wire because they presented a lower fracture. Overall 6/81 patients were lost at follow up.

Exclusion criteria were: associated proximal intra-articular or distal intra-articular fractures of the tibia, tibial plafond fractures, vascular injury requiring repair, pathologic fractures, previous fractures of the same limb, open fractures along with past or present corticosteroid use.

All of the fractures: 75 were classified with AO system and the obtained results are described in Graph 1.

**Figure 5:**
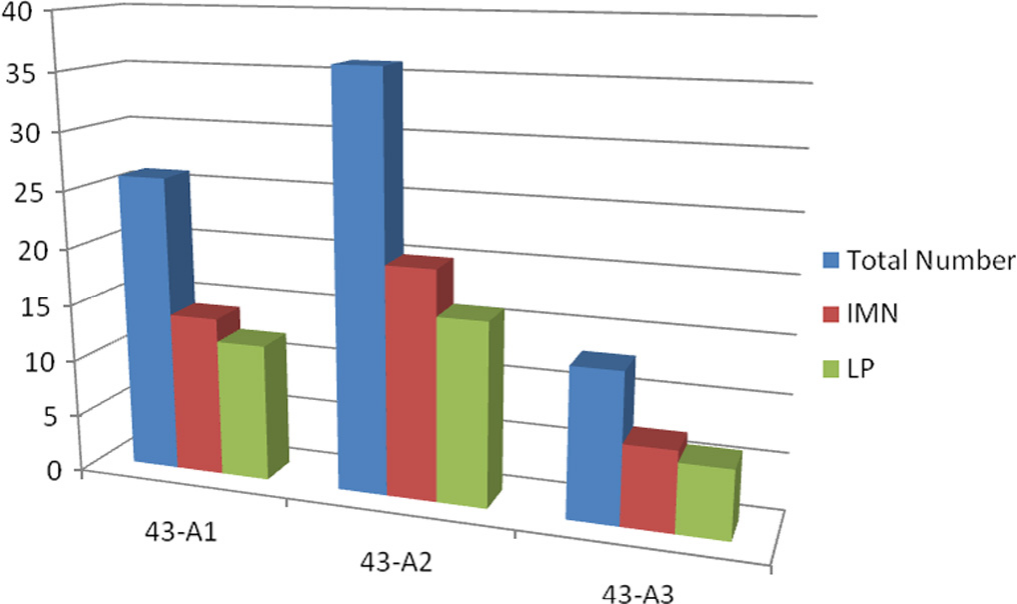
Subdivision of the 75 fractures according A.O. Trauma classification.

- 14 with a 43-A1 fracture in the IMN group and 12 in the LP group;
- 20 with a 43-A2 fracture in the IMN group and 16 in the LP group;
- 7 with a 43-A3 fracture in the IMN group and 6 in the LP group.

The overall mean distance of the fracture from the joint was 80.1 mm (range 40–96.5 mm): IMN group: 73.2 (range 50.5–96.5 mm); LP group: 59.2 (range 40–53.8).

In the IMN group we used Trigen Smit and Nephew nails with parapatellar medial access. While in the LP group, we used and antero-medial access.

There were 27 female and 48 male patients. In 38 cases, the right limb was involved while in the remaining 37 cases the left limb was involved. Mean age was 31 years (range 18–71 years).

**Figure 6:**
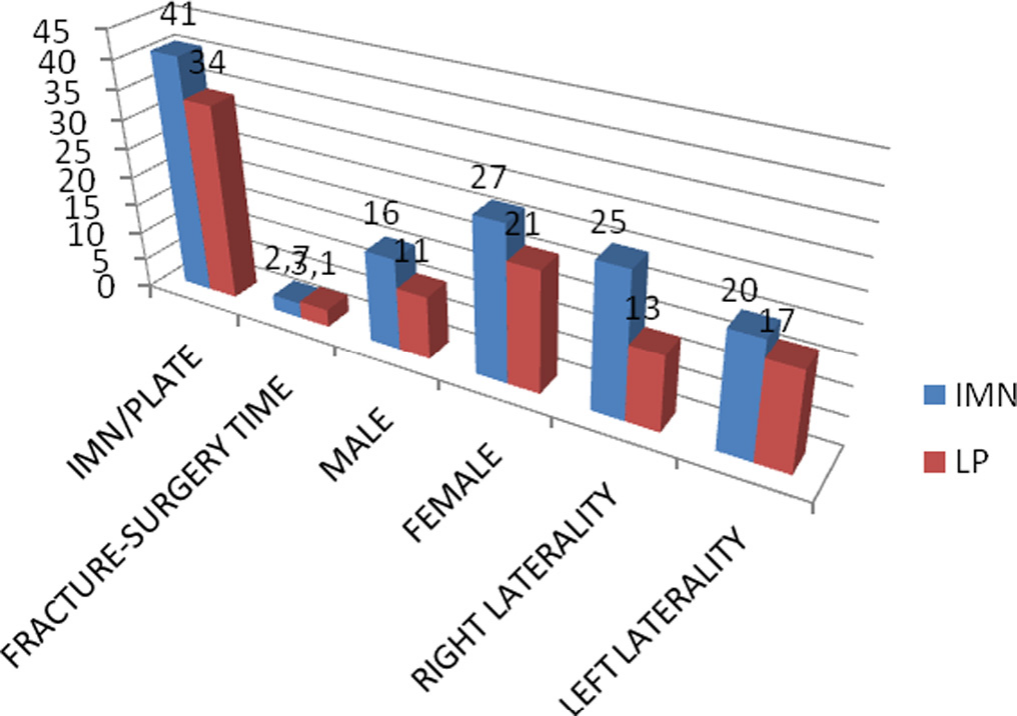
Description of the population.

Age, sex, time between fracture and surgery along with side of the fracture are described in Figure 6.

In 72 patients, we performed a close reduction of the fracture and temporarily applied a open plaster. A skeletal traction was applied in 9 cases.

All patients received 4000 international units of enoxaparin sodium to prevent thromboembolism and 2 g of intravenous cefazolin as preventive antibiotic therapy before the operation. All surgical procedures were performed using bi-block anesthesia on a radiolucent operating table.

**Figure 7:**
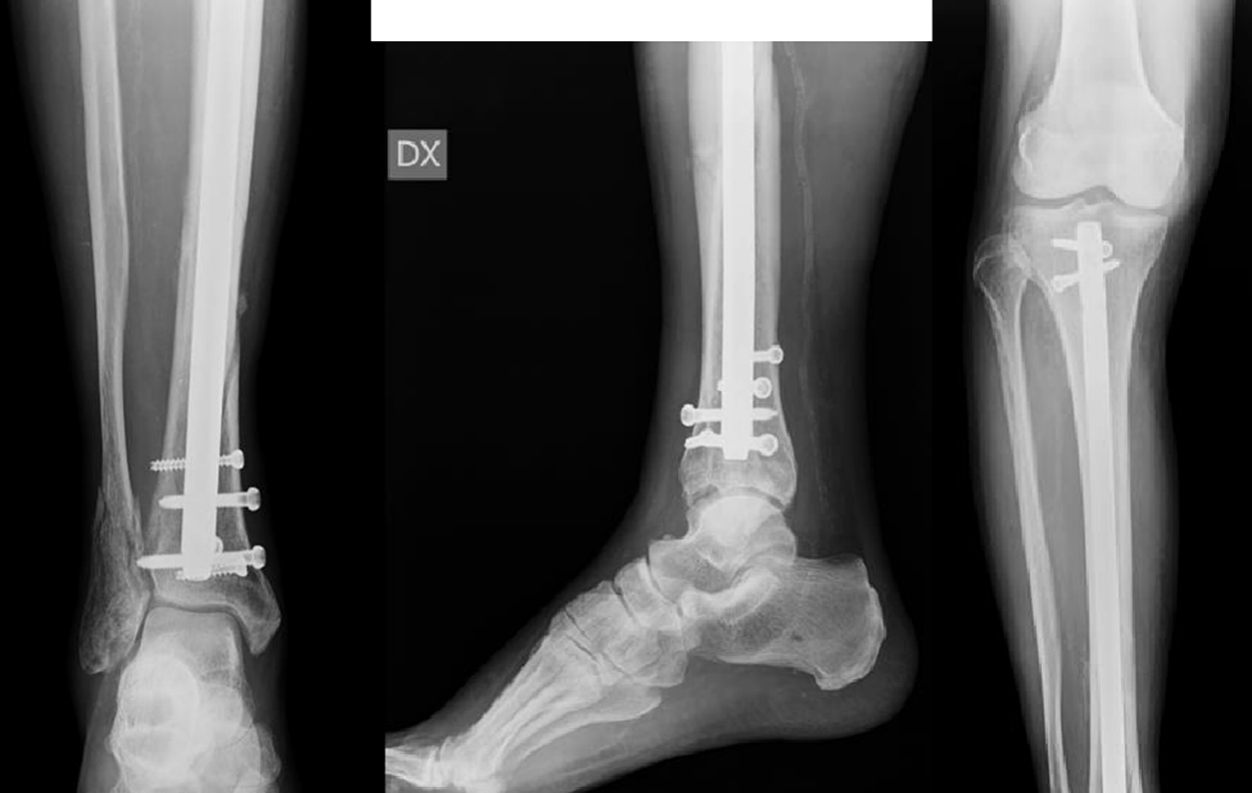
(a,b,c) Post-operative radiography at 3 months follow-up of IMN.

**Figure 8:**
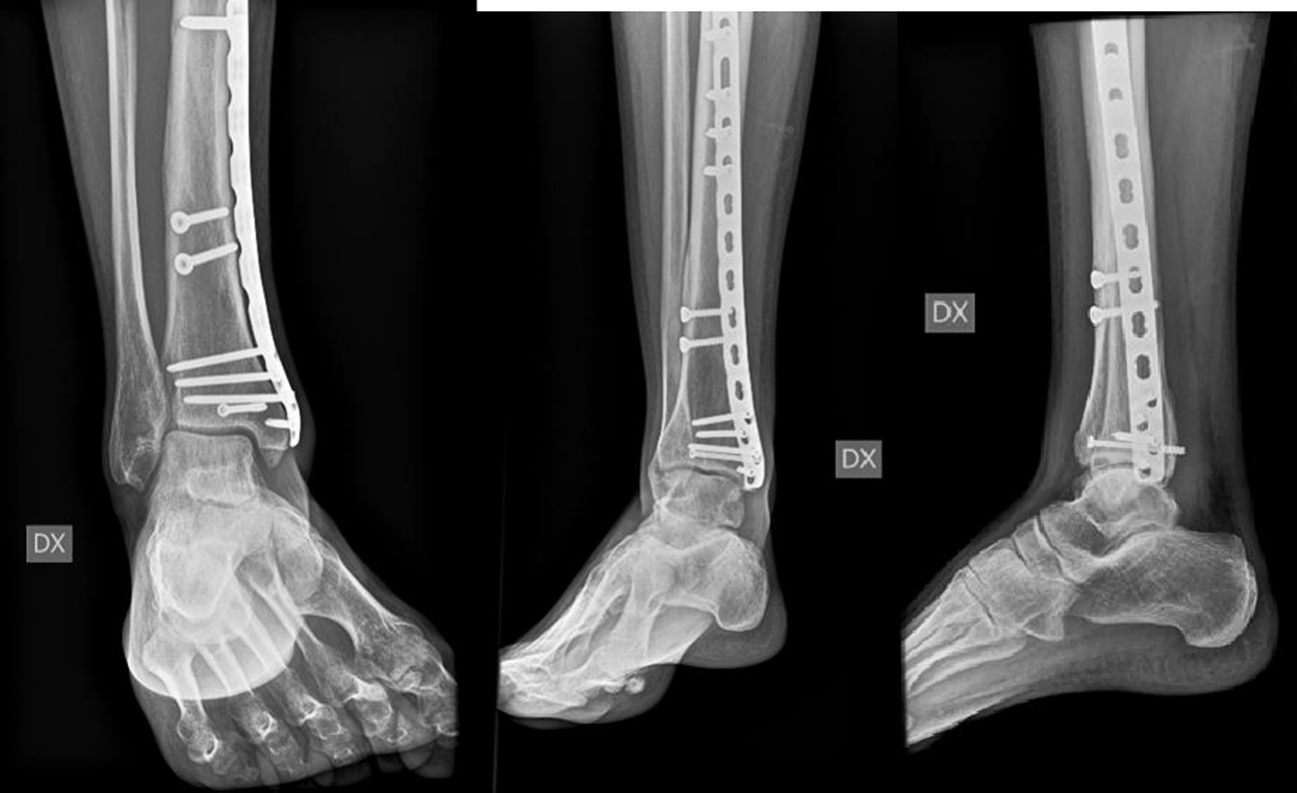
(a,b,c) Post-operative radiography at 6 months follow-up of LP patient with good formation of bone callus.

Clinical and radiological follow-ups were done at 1, 3, 6 and 12 months after the operation (Figures 7 and 8).

Nonunion was defined as the absence of any sign of callus formation after 6 months. Moreover, malunion was defined as angulation of more than 5° on any plane. Union was clinically defined as the ability to walk without pain and when a radiograph showed a solid bridging callus of obliteration of the fracture line. Radiological assessment was performed in antero-posterior and lateral views.

Our patients were encouraged to perform ankle flexion and extension exercises after the operation; partial-weight bearing was allowed after three weeks in both groups.

Clinical results were assessed using the Olerud–Molander Ankle Score [11,13–15].

### Statistical analysis

We used Student's t test to compare the inter-group parameters with quantitative data and descriptive statistical methods (mean, standard, frequency). We used the chi-square test and Fischer's exact chi-square test to compare qualitative data. The significance level was set at *p* < 0.05.

## Results

Overall, the complete case series included 75 patients.

The mean time between the trauma and the operation was 2.7 days for the IMN group (range: 1–6) and 3.1 for the LP (range 1–7); without being statistically significant.

The mean hospital time after surgery was 4.5 days for the IMN group (range 3–7 days) versus 5 days for the LP group (range 3–8 days); without resulting statistically significant (*p* < 0.05).

The mean operation time was 78 min for the IMN group (range 75'–83') and 92 min for the LP group (range 88'–97'). This difference did not result being statistically significant (*p *> 0.05).

The mean union time was 21.8 weeks for the IMN group (17.4–23.3) and 24.2 weeks for the LP group (range 17.6–28.3). This difference did not result being statistically significant (*p *> 0.05).

The infection rate for the IMN was 0; while the same rate was 5.88% for the LP group (2 patients developed infection). This rate difference was not statistically significant (*p *> 0.05). The two infected patients were treated by removing the synthesis means, implanting a temporary external fixation and adequate antibiotic therapy was prescribed. After the infection was healed, we performed a new surgical procedure to implant a second locked plate.

In the IMN group, 9 patients developed a malunion (rate 21.9%): 6 varus and 3 valgus deformities. Thus, we did not obtain a malunion greater than 11°. In the LP group, no patient developed a malunion (rate 0%). This rate difference resulted being statistically significant (*p *> 0.05).

No patient in the two groups developed a nonunion.

In the IMN group 8 patients developed anterior knee pain (19.5%). This rate was higher than those reported in previously published clinical studies; probably depending on the nailing approach. Indeed, with the trans-patellar access, pain could be due to patellar tendon and retro-patellar fat pad damage [5]. We used only the para-patellar approach.

The full weight bearing time was significantly longer in the LP group compared to the IMN group (15.3 ± 2.9 weeks versus 12.8 ± 3 weeks, respectively). This difference was statistically significant (*p* < 0.05).

Olerud–Molander Ankle Score: 30 patients in the IMN group and 22 patients in the LP group obtained an excellent outcome (rate 62.2% and 64.7%, respectively), 11 patients in the IMN group and 12 in the LP group obtained a good outcome (rate 26.8% and 35.3%, respectively). None of the patients obtained a fair or poor outcome. Overall 52 patients obtained an excellent result (69.3%) and 23 obtained a good result (30.6%).

## Discussion

In the context of distal tibial fractures, surrounding soft tissues are often damaged; therefore, a treatment that respects these tissues is very important. A complete reduction of the fracture could be obtained with an anatomical plating, that require large incisions and, subsequently, a risk of high rate of infections and tissue suffering, while minimal invasive methods minimize the damage of the soft tissues [13,14]. At present, the main surgical procedures for the treatment of tibial distal fractures are intramedullary nails, locked plates and external fixation. The latter procedure is particularly indicated when the cutaneous suffering determined by the high-energy traumatic event does not allow any other surgical procedure [7,8–10,15].

Several studies were carried out [16,17] to compare intramedullary nailing to plating, plating to external fixation and intramedullary nailing to external fixation. The aim of our study was to compare IMN to LP [18,19]. Actually, at present, their indications are still discussed.

Various clinical studies have compared IMN and LP [13,20–22]: the former leads to a lower rate of soft tissue complications and infections and has been associated with a significantly shorter full weight bearing and a shorter union time. On the other hand, IMN appears has been reported to lead to a higher rate of malunion and nonunion because it may involve reduction issues [16,19,20,23].

Locked plate is advantageous given that it generally leads to a better and a greater reduction of the fracture. Additionally it allows for a better stabilization of distal tibial fractures and it advances the bone healing more than intramedullary nails [23–28].

In our study, we did not find a statistically significant difference in terms of operation time, hospital stay, infection rate, union time and functional outcomes between the two groups but we did observe at least 3 points of interest. First, regarding the rate difference in terms of malunion, 9 patients developed a malunion in the IMN group (rate 21.9%) while no patient developed a malunion in the LP group (rate 0%). This event could occur because locked plating advances a more anatomical and fixed reduction of the fracture, while intramedullary nailing treatment mainly permits minimal movements of the bone fragments.

Second, regarding the time of permission to apply full weight bearing, it was 12.8 ± 3 weeks for the IMN group, and 15.3 ± 2.9 weeks for the LP group. We allowed full weight bearing depending on the operator surgeon indications, based on clinical and radiographic signs. This suggests that intramedullary nailing guarantees a significantly shorter full weight bearing time than locked plating.

Finally, with regards to functional outcome patients in the two groups had similar Olerud–Molander Ankle Scores: 30 in the IMN group and 22 in the LP group obtained excellent outcomes, 11 in the IMN group and 12 in the LP group obtained good outcomes; none of the 75 patients obtained a fair or poor outcome.

We did not obtained significant results in terms of union time. Probably in the LP group union was advanced by a more anatomical reduction, in the IMN group it was advanced by a shorter bearing time.

These results strongly suggests that intramedullary nailing and locked plating treatment are comparable treatments when considering functional outcome for distal tibial fractures. Our study results indicate a superiority of IMN over LP in terms of lower rates of infections and statistically significant shorter time to full weight bearing. Whereas LP appeared to be advantageous over IMN in terms of leading to a better anatomical and fixed reductions of the fracture and a lower rate of union complications. The two treatments achieved comparable results in terms of operation time, hospital stay, union time and functional outcomes. A future clinical study will need to include at least 300 patients in order to better characterize any differences or similarities between IMN and LP in patients with distal tibial fractures.

## Conflict of interest

The authors declare no conflict of interest.
